# Seroprevalence of dengue, yellow fever, and related flaviviruses among the rural human population in Nguruman and Kerio Valley, Kenya

**DOI:** 10.3389/fviro.2024.1459021

**Published:** 2024-09-24

**Authors:** Mercy Hokah Kibathi, Edith Chepkorir, Sepha Nyatichi Mabeya, David P. Tchouassi, Rosemary Sang

**Affiliations:** 1 Human Health Division, International Centre of Insect Physiology and Ecology (ICIPE), Nairobi, Kenya; 2 Department of Medical Microbiology, Jomo Kenyatta University of Science and Technology (JKUAT), Nairobi, Kenya; 3 Centre for Virus Research, Kenya Medical Research Institute (KEMRI), Nairobi, Kenya

**Keywords:** arbovirus surveillance, flaviviruses, yellow fever virus, dengue virus, plaque reduction neutralization test, Zika virus, West Nile virus, seroprevalence

## Abstract

**Background:**

Yellow fever virus (YFV) and dengue virus (DENV) are among the major re-emerging arboviruses that pose a significant threat to public health. Their associated burden and prevalence can be substantially underestimated due to insufficient surveillance and inadequate diagnosis. This study aimed to determine evidence of dengue, yellow and related flaviviruses circulation among the rural human populations residing in Nguruman (Kajiado County) and Kerio Valley (Baringo County), two dryland ecosystems in the Kenyan Rift Valley.

**Methods:**

Serum samples obtained from febrile patients between 5 and 85 years through a hospital-based cross-sectional survey from July 2020 – May 2023, were screened for neutralizing antibodies to YFV, DENV-2 and related flaviviruses, West Nile virus (WNV) and Zika virus (ZIKV) via Plaque reduction neutralization test (PRNT). The study sites and important demographic characteristics were obtained using a structural questionnaire and the data analyzed and seroprevalence compared. A multinomial logistic regression model was done to predict risk for each of the most prevalent viruses with covariates; age, gender, and occupation.

**Results:**

Overall, 54.5% (50.1–59.0% 95% confidence interval (CI) of the samples tested positive for at least one of the four Flaviviruses. The percentage was significantly higher in Kerio Valley (64.34%, 184/286) than in Nguruman (40.2%, 78/194) (P<0.0001). YFV had the highest prevalence, followed by WNV (16.25%), ZIKV (5.2%), and DENV-2 (1%). Kerio Valley had a significantly higher YFV seroprevalence (51%) than Nguruman (6%) (P<0.0001), while DENV-2 was observed only in Nguruman with a low seropositivity of 2%. In contrast to Nguruman, where seropositivity rates were higher in males at 47.47% (P=0.049), in Kerio Valley, females showed considerably higher viral seropositivity at 60.82% than males (P<0001).

**Conclusion:**

The study suggests that there is significant circulation of Flaviviruses in both regions, posing a public health risk, that could potentially contribute to clinical disease. However, seropositivity rates vary for each specific site. Furthermore, there could be a risk of YFV, WNV, and ZIKV transmission in both sites with DENV transmission specifically noted in Nguruman. The study findings inform direct cost-effective actions (such as YF vaccines) and precise surveillance data of vector populations for improved disease risk prediction.

## 1 Introduction

Dengue virus (DENV) and yellow fever virus (YFV) belong to the family *Flaviviridae* and genus *Flavivirus* and cause significant morbidity, mortality, and economic burden, especially in Sub-Saharan Africa (1–4). They are mostly endemic in tropical and subtropical regions (5). These illnesses share a similar ecological niche, with nonhuman primates serving as reservoir hosts, and are largely transmitted by *Aedes* subgenus *Stegomyia* mosquitoes (6, 7).

Dengue virus is the world’s fastest-spreading arbovirus with nearly half of the global population (about 4 billion people) now at risk of contracting dengue fever, and more particularly in tropical and subtropical regions (3). An unanticipated surge in dengue infections coupled with continuous transmission since the start of 2023 has led to a record high of over 6.5 million illnesses and over 7300 dengue-related deaths recorded, indicating a widening epidemiological scope (3, 6). Dengue virus occurs in four closely related, but distinct serotypes (DENV 1 – 4) (8, 9), each causing dengue fever. More than 80% of human dengue cases manifest as minor acute flu-like diseases, with around 5–10% of human dengue cases developing into severe dengue, which is characterized by dengue shock syndrome and hemorrhagic fever (3, 10, 11).

Dengue fever virus was first detected in Kenya during an outbreak in the coastal region of Mombasa, Kilifi, and Malindi in 1982 (12) followed by another outbreak in Mandera, North Eastern Kenya, close to three decades later in 2011, that subsequently spread to the Kenyan coast (13–15). Kenya has documented cases of all four dengue viral serotypes 1 – 4, with the dengue 2 virus being the most common serotype (15, 16). This geographic dengue outbreak trend in Kenya is of concern as it shows the potential for further transmission and spread (7).

Yellow fever virus was first detected in Kenya in Kerio Valley, Baringo County in 1992–93 (17, 18). The bulk of YF cases occur in West and Sub-Saharan Africa, where 33 nations and an estimated more than 900 million people live in YFV endemic regions, accounting for roughly 90% of total global infections that result in 30,000 to 200,000 cases each year (19, 20). The recent YFV outbreak in Central and East Africa occurred in 2015 in urban areas of Angola’s Luanda Province, resulting in over 7000 cases and 500 deaths before spreading to Kinshasa (DRC) by July 2016 (7, 21). Kenya has been categorized as being at high risk for YF transmission and outbreaks according to the Eliminate Yellow Fever Epidemics Strategy (EYE) by the World Health Organization (22). Furthermore, the central part of Kenya, Isiolo Counties recorded a total of 53 probable yellow fever cases and six mortalities in March 2022. From suspected cases, six were confirmed using Enzyme-linked immunoassay (ELISA) and Plaque reduction neutralization assay, with reverse transcription polymerase chain reaction (RT-PCR) confirming two cases, suggesting possible cases of yellow fever (23). Given the recent recurring YFV outbreaks in the neighboring nations of Uganda, South Sudan, and Ethiopia (2, 24–27), there is potential for cross-border spill-over via migration of infected non-human primates, people, or mosquitoes.

The related *Flavivirus*, West Nile virus (WNV) a zoonotic mosquito-borne virus initially isolated in Uganda in 1947 is currently re-emerging with a geographic range and frequency continually expanding (2, 28, 29). Zika virus (ZIKV), on the other hand, was discovered in the Zika Forest, Uganda in 1947 after being isolated from a febrile monkey. It was later found in *Aedes africanus* mosquitoes collected from the same forest, and human cases were found there in 1962–1963 (7, 30). The first records of the ZIKV virus and humans date back to 1954, when three cases in Nigeria with fever and jaundice were found (31). Ever since, human serosurveillance studies have indicated that the Zika virus is present in Asia, Oceania, and Africa (30–32). According to a recent investigation of samples collected during the dengue outbreak in 2013, ZIKV co-circulated in Kenya (33). In addition, another study discovered that up to 7% of Northern Kenyans had neutralizing anti-ZIKV antibodies (2). Following insufficient surveillance, the actual ZIKV burden in Kenya remains poorly known.

The increasing frequency and geographic expansion of these arboviruses in recent decades in Africa remains a concerning threat (21). These increasing trends of cases connected to the rapidly increasing human population, fast-expanding urbanization without sufficient sanitary infrastructure, increased international travel, deforestation, and climate change call for public health intervention (4, 34).

Epidemiologic and entomological data on the prevalence and distribution of these *Flaviviruses* in Nguruman and Kerio Valley are scarce. The low level of clinical awareness and inadequate diagnostic capabilities limit disease recognition and detection of cases at the facility level (7). This problem is made worse by the coexistence of other endemic diseases like leptospirosis, brucellosis, and malaria and by the limited capacity for differential diagnoses in facilities with inadequate health infrastructure. Consequently, the extent of exposure and the prevalence of these serious *Flavivirus* infections in these areas are still poorly understood. Thus, this study sought to determine evidence of dengue, yellow fever, and related Flaviviruses among the rural human population residing in Nguruman and Kerio Valley, Kenya.

## 2 Materials and methods

### 2.1 Ethical approval

The study was granted approval by the National Commission for Science, Technology & Innovation under Research license number NACOSTI/P/23/25147 and the Scientific Ethics and Review Unit (SERU) of the Kenya Medical Research Institute (KEMRI) under protocol number KEMRI/SERU/CVR/013/4664. Written informed consent was given by each adult participant, and minors’ or children’s assent was obtained. In the study, only those who gave their assent or consent were included.

### 2.2 Study design, sites, and population

**FIGURE 1:**
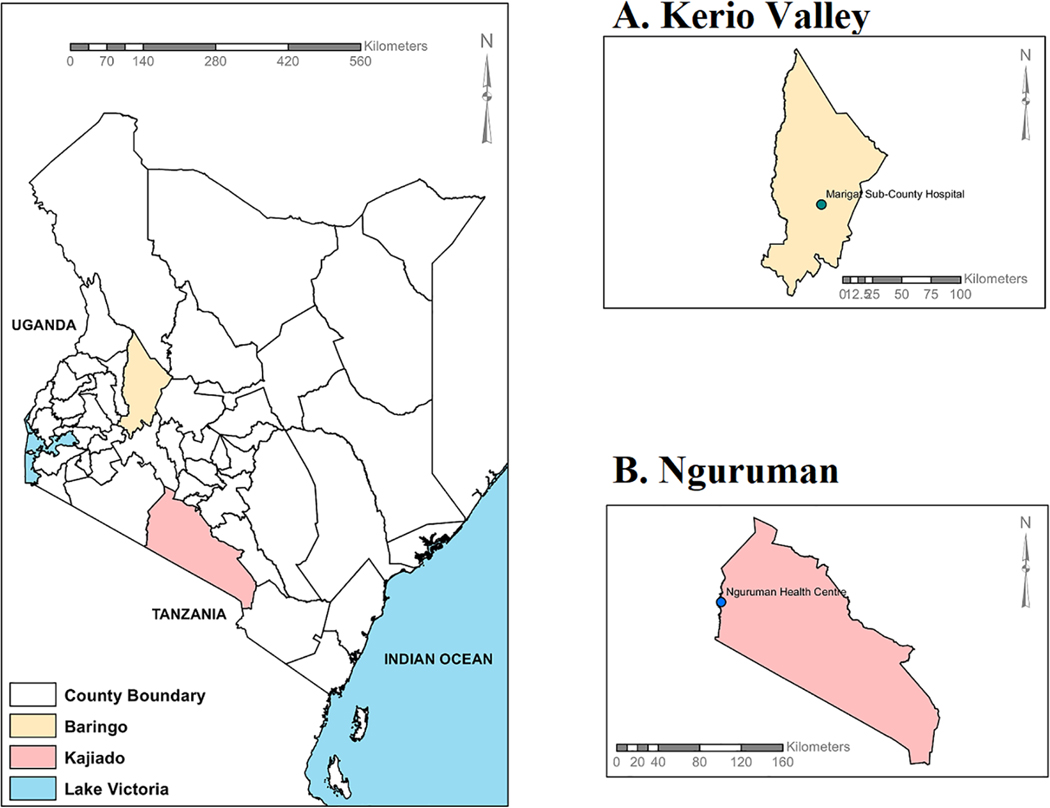
Kenyan map displaying the locations of the two study sites: (**A**) Kerio Valley and (**B**) Nguruman. The maps were created with ArcGIS Desktop Version 10.2.2 (Advanced License).

The study was a cross-sectional hospital-based serosurvey that leveraged an acute febrile illness surveillance sampling of patients presenting with febrile illness (a body temperature >37.5 degrees Celsius, confirmed negative for malaria, typhoid and brucellosis) who were identified and sampled by local clinical staff at the Nguruman Health Centre in Nguruman town, and in Marigat Sub-County hospital, in Kerio Valley (Figure 1). The human population in both Nguruman and Kerio Valley are mostly pastoralists and occasionally farmers. In 2019, 666, 763 people lived in Baringo and 1,117,840 people in Kajiado (35). Both Nguruman and Kerio Valley have a similar dryland ecosystem occupied by largely pastoralist communities, diverse livestock and wildlife species with past evidence of arboviral circulation (36–38). Low-lying plains and seasonal rivers provide grazing land, water for irrigation, food for animals and wildlife, and a means of subsistence for the human population. Both sites have dry African savannah terrain, with low-lying Acacia plants, discontinuous trees and long grasses predominating. While Kerio Valley in Baringo County is around 250 kilometers northwest of Nairobi, Nguruman in Kajiado County is situated in southern Kenya and borders Tanzania (Figure 1). Unlike Nguruman which has never experienced a YFV outbreak nor YFV vaccination, Kerio Valley experienced an outbreak of YFV in 1992/93 which was brought under control by mass vaccination with the subsequent institution of routine YF immunization for all children at 9 months in Kerio Valley to avert future outbreaks (17, 18). Based on the information given during sampling, the samples tested from Kerio Valley in Baringo County were from participants who were not vaccinated. However, records of vaccination status were based on self-reporting which could not be verified, as no vaccination cards were provided during the sample collection. However, following the 1992/93 YFV outbreak in Rift Valley, routine vaccination was instituted in Baringo County. Most of the participants may not remember being vaccinated.

### 2.3 Sample collection

An estimated 5ml of venous blood sample was obtained during a cross-sectional hospital-based survey from febrile consenting participants visiting the selected health facilities during the period between July 2020 and May 2023. Blood was separated and serum kept in a liquid nitrogen shipper at the site and transferred to the International Centre of Insect Physiology and Ecology (ICIPE) for further laboratory analysis. Both males and females aged between 5 and 85 years old presenting with a malaria-like fever illness were recruited into the study. A structural questionnaire was administered whereby demographic data on; age, occupation, gender, status of yellow fever vaccination, residence places and any relevant history of travel by participants was obtained.

### 2.4 Viruses and cell lines

Although the focus was on yellow fever and dengue-2 viruses, testing for neutralizing antibodies for the most common *Flaviviruses*; dengue, yellow fever, Zika, and West Nile viruses, was performed by PRNT to rule out cross reactivity. Dengue-2 was chosen because it is the most common dengue serotype in Kenya (12, 14, 15). The neutralization was done using live preserved viral isolates (DENV-2 008/01/2012, YFV YFXSMB, ZIKV MR766, and WNV AMH005348) obtained from KEMRI’s Viral Hemorrhagic Laboratory (VHF). To conduct virus titration and neutralization testing, the viruses were propagated in the C6/36 (Aedes albopictus mosquito) cell lines and inoculated on Vero cell lines (CCL81, and E6).

The inoculated viruses were harvested and quantified using plaque assay as described by (39). Briefly, ten-fold serial dilutions of the viral stocks were prepared. 2.0 ml microcentrifuge tubes (Eppendorf, USA) were labelled as 10^−1^, 10^−2^, 10^−3^, 10^−4^, 10^−5^, 10^−6^ and Negative. 900ml of maintenance media (Minimum Essential Media Eagles, Sigma, enhanced with 2% heat-inactivated fetal bovine serum (FBS), 2% penicillin/amphotericin B, and 2% L-glutamine (Sigma-Aldrich, St. Louis, MO) was added to each tube. The viruses were serially diluted 10-fold by transferring 100 ml of each virus to the appropriate tube labelled 10^−1^, vortexing, and transferring 100μl to the subsequent tube, down to 10^−6^ with the last 100μl being discarded. Subsequently, Vero cells (E6) were seeded into a 12-well plate (Corning, USA) and incubated for 24 hours in a 5% CO2 set at 37°C to allow the formation of a confluent monolayer. Next, the plate was labelled properly, and 100μl of the diluted virus (10^−1^, 10^−2^, 10^−3^, 10^−4^, 10^−5^, and 10^−6^) was dispensed into the specified wells, with each pipette tip being changed. A negative control was included in the test. For one hour, the plate was incubated in 5% CO_2_ at 37°C, with 15-minute intervals of rocking to facilitate virus adsorption. After adsorption, the cells were overlaid with 1ml methylcellulose (2.5%) (Sigma) mixed with 2X Minimum Essential Medium (Sigma). The well plates were placed in a 5% CO_2_ set at 37°C. Following a period of 6 to 14 days, depending on the virus under investigation, the plates were fixed in formalin and stained using crystal violet (diluted in 100% ethanol) for visualization of plaques. The plaques formed were manually counted and calculated to quantify the virus using the formula below (40).

$$\frac{\mathrm{Number}\;\mathrm{of}\;\mathrm{plaques}}{d\times V}=\mathrm{pfu}/\mathrm{ml}$$

where **d** is the dilution factor and V is the volume of diluted virus added to the well.

Viral titer was expressed as plaque-forming units (pfu) per ml and an appropriate virus dilution factor (containing 20–70 plaques) (39) was used to calculate the amount of virus needed for dilution of the virus to be used for PRNT. The calculated viral titers for the viruses used are shown in Table 1.

### 2.5 Plaque reduction neutralization test

Serum samples were removed from the −80°C freezer, thawed, aliquoted in 30ml volumes, and heat-inactivated for 30 minutes at 56°C. Using the Plaque Reduction Neutralization Test (PRNT_90_), the samples were examined for neutralizing antibodies to the DENV-2, YFV, WNV, and ZIKV viruses in two-fold serum dilutions ranging from 1:10 to 1:5160. The viruses were amplified in cell culture, tittered by plaque assay, and working stock diluted to 20 – 70 plaques per 50ml. Beginning with a seropositivity threshold of 1:10 ten-fold dilutions the serum was prepared using the maintenance media (Minimum Essential Media Eagles, Sigma, enhanced with 2% penicillin/amphotericin B, 2% heat-inactivated fetal bovine serum (FBS), and 2% L-glutamine (Sigma-Aldrich, St. Louis, MO). The diluted serum sample was mixed with 30ml of the known constant concentration of the diluted virus in microcentrifuge tubes followed by a 1-hour incubation at 37°C. The virus-antibody mixture was added to a 24-well plate that contained a confluent monolayer of VERO E6 cell lines. The plates were then incubated for one hour at 37°C in an incubator with 5% CO2 to allow the virus to adsorb. After 1 hour, each well was maintained with 1ml methylcellulose (2.5%) (Sigma) overlay mixed with 2X Minimum Essential Medium (Sigma). After 6 to 14 days, subject to the virus being examined, the plates were fixed with formalin, stained with crystal violet (diluted in absolute ethanol) and plaques counted manually (2, 34, 41). Plaque reduction neutralization test (PRNT) end-point titers (seropositivity threshold) are expressed as the reciprocal of the last serum dilution showing the desired per cent reduction in plaque counts (42). In our study, we reported the endpoint titer as the reciprocal of the highest serum dilution that resulted in ≥ 90% reduction in plaque counts (PRNT_90_) (43). Thus, PRNT_90_ was used to calculate the highest serum dilution (1:10 to 1:5160) required to reduce DENV-2, YFV, WNV and ZIKV plaque formation by 90% in Vero cells (44). In cases where two or more viruses neutralized the same sera sample, based on the WHO guidelines and criteria, the virus with an antibody titer of four-fold or higher than the other flaviviruses tested was considered viral-specific and hence positive (42, 45–47).

### 2.6 Statistical analysis

All the data collected was stored in password-protected folders and analyzed using R software version 4.3.2 (48) and Microsoft Excel. PRNT endpoint titer was computed as the reciprocal of the last serum dilution demonstrating the intended per cent reduction in plaque counts. Thus, the endpoint titer was reported as the reciprocal of the highest serum dilution that produced a reduction in plaque counts of at least 90% (PRNT_90_) (42, 43). The participants were first characterized based on site and demographic characteristics. Descriptive statistics were performed and the prevalence was compared by site and demographic characteristics i.e., gender, age and occupation. The heterogeneity of the seropositive proportions was evaluated and proportions were compared using the Chi-Square test. A multinomial logistic regression model was used to predict risks for each of the most prevalent viruses with covariates in association with age, gender, and occupation data. The odds ratio (OR) generated was used to assess the association between the risk factors and the outcomes. A p-value of 0.05 or less was considered significant at a 95% confidence level.

## 3 Results

### 3.1 Demographic characteristics of the study participants

The study participants’ demographic characteristics for both the individual and combined study sites are displayed in Table 2. This study population comprised a total of 480 participants, 194 (40.4%) from Nguruman and 286 (59.6%) from Kerio Valley. 60.21% (289/480) were females and 39.79% (191/480) were males. The sampled participants’ ages ranged from 5 to 85 years with a mean and median age of 27 years and 24 years respectively. About 37.16% of the participants were farmers, 30.48% were students, 5.64 were businesspeople. 16.08% were housewives, while 1.67% engaged in other economic activities and 8.98% had no indicated occupation. Participants were structured into four age groups; Below 25 years, 26–45, 46–65, and 66 and above. Most of the participants (56.25%) were below 25 years which comprised mostly school-going children. All the participants indicated that they were not vaccinated against the yellow fever virus during the survey.

### 3.2 Prevalence of neutralizing antibodies against Yellow fever, Dengue-2, and related Flaviviruses in Nguruman and Kerio Valley

**FIGURE 2:**
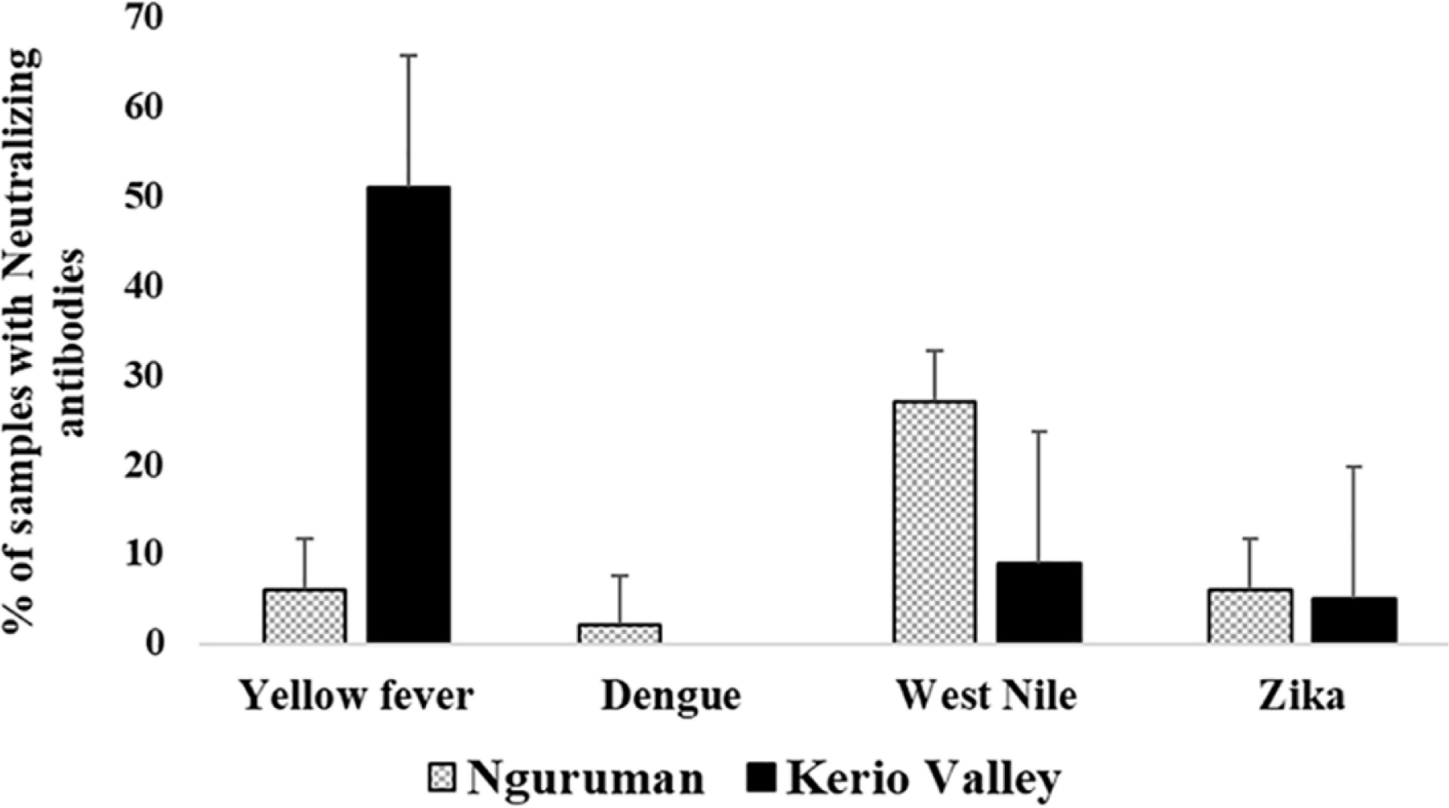
Seroprevalence of neutralizing antibodies against yellow fever, dengue-2, and related flaviviruses categorized by study sites. The number of samples tested was n=286 in Kerio Valley and n=194 in Nguruman, the error bars indicate a 95% confidence interval.

Overall, 54.5% (262/480; 95% CI, 50.1–59.0%) of the samples had neutralizing antibodies to at least one of the four flaviviruses screened with higher proportions being detected in in Kerio Valley (64.34%, 184/286) than in Nguruman (40.2%, 78/194) (P<0.0001). From viral neutralization, the seroprevalence of the neutralizing antibodies categorized according to study sites showed that overall, yellow fever virus (32.5%) had the highest prevalence of neutralizing antibodies followed by West Nile (16.25%), Zika (5.2%), and Dengue-2 (1%) viruses (Table 3). In addition, the seroprevalence of the neutralizing antibodies against flaviviruses varied across the sites. Kerio Valley had a significantly higher YFV seropositivity (51%) than Nguruman (6%) (P<0.0001), while Dengue-2 was observed only in Nguruman with a low seropositivity of 2%. Whereas the rates of ZIKV were comparable, at 5% (13/286) in Kerio Valley and 6% (12/194) in Nguruman, WNV exposure rate was significantly greater in Nguruman (27%, 52/194) than in Kerio Valley (9%, 26/286), (P<0.0001) (Table 3; Figure 2).

**FIGURE 3:**
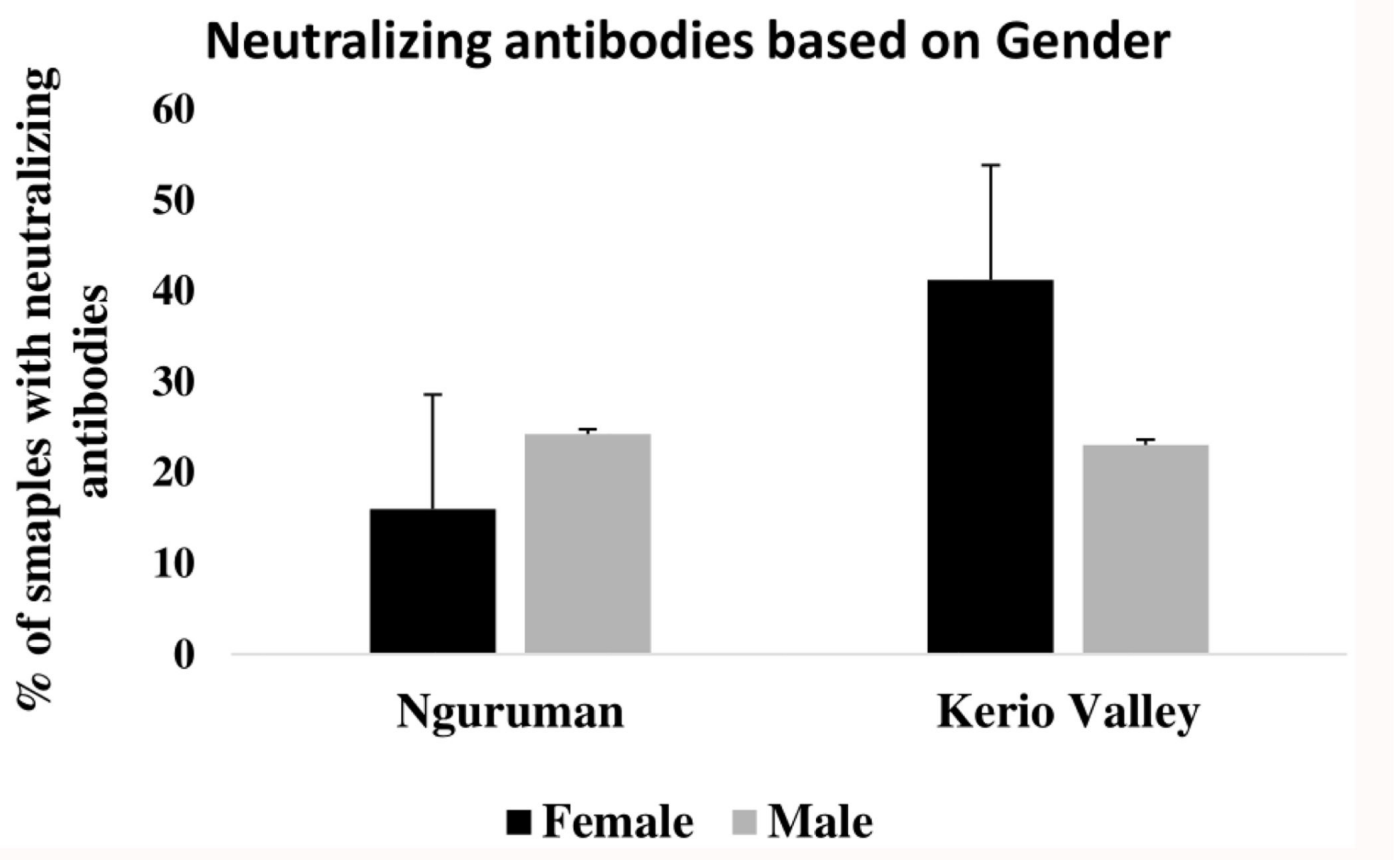
Proportion of samples with flavivirus neutralizing antibodies based on gender, for both Nguruman and Kerio Valley, the error bars indicate a 95% confidence interval.

Figure 3 presents the seroprevalence of neutralizing antibodies for both study sites based on gender. The findings indicate that in Kerio Valley, seropositivity was significantly higher in females (118/286; 41.26%) than in males (66/286; 23.08%) (P<0001). In Nguruman, however, males’ seropositivity rates were higher (47/194; 24.23%; P=0.049).

**FIGURE 4:**
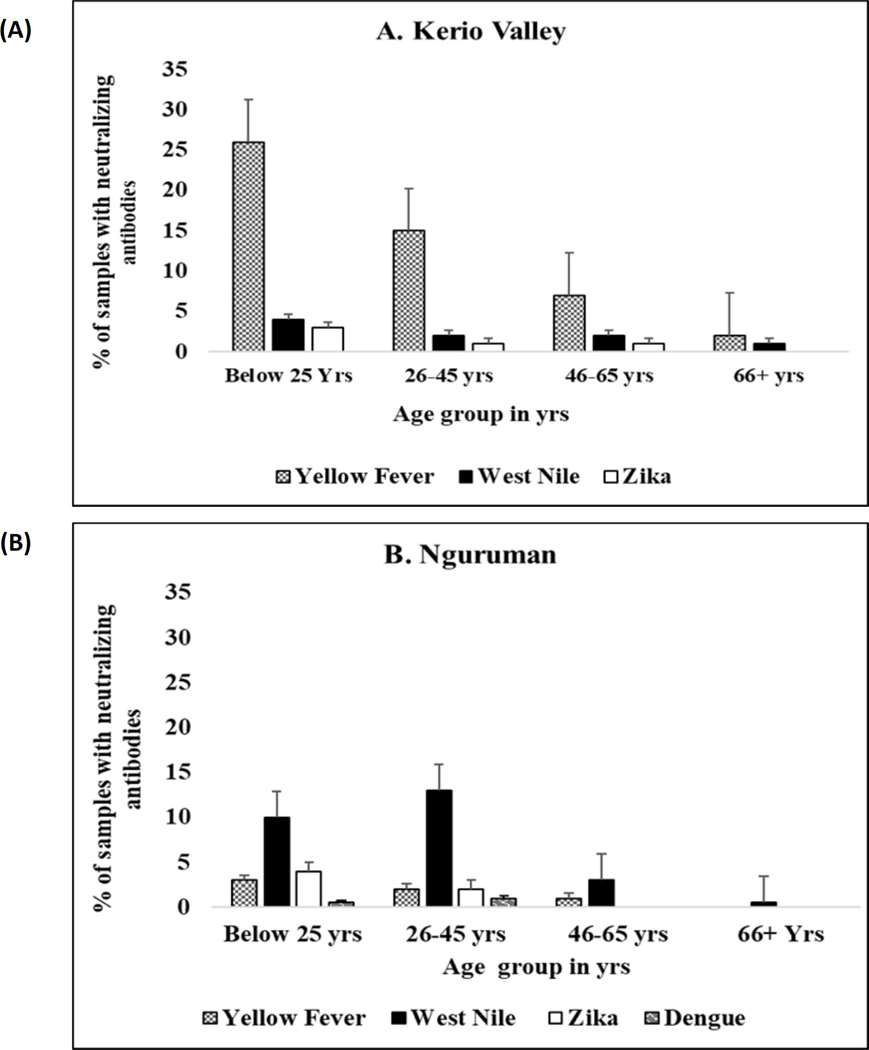
Proportion of samples with flavivirus neutralizing antibodies by age group in (A) Kerio Valley, n=286 and (B) Nguruman, n =194, the error bars indicate a 95% confidence interval.

Seroprevalence of neutralizing antibodies against the four flaviviruses also varied by age group (Figure 4). In Kerio Valley, the prevalence of YFV-neutralizing antibodies was highest in the below 25 years’ age group and lowest in the 66+ years age group, a similar pattern was observed for WNV and ZIKV neutralizing antibodies, with the highest prevalence among the below 25 years’ group and the lowest in the age group 66+ years. In Nguruman, age was significantly associated with WNV seropositivity (P= 0.02) being highest among the age group 26 – 45 years and lowest in the age group 66+ years while DENV-2 neutralizing antibodies’ prevalence was highest in the age group 25 and below and lowest in the age group 26 – 45 years.

### 3.3 Demographic predictors for seroprevalence of the most prevalent Flaviviruses in Kerio Valley and Nguruman

Table 4 shows results estimated by the Multinomial Logistic Regression model. The findings show that in Kerio Valley, men were approximately two times more likely to have been exposed to YFV than females (OR =1.31, 95% CI = 0.26–6.50). There were no significant differences between yellow fever virus prevalence and participants’ age and occupation in Kerio Valley. Males were twice more likely to be seropositive than females for WNV in Kerio Valley (OR= 2.19, 95% CI=1.14–4.19). Likewise, WNV seroprevalence was about three times more in the age group 26–45 years than below 25 years (OR = 2.83, 95% CI=1.13–7.12, P value = 0.027). The occupation did not affect WNV seroprevalence. In Nguruman, the likelihood of WNV exposure was about 2.49 times higher in males compared to females (OR =2.49, 95% CI = 1.40–4.42, P=0.002), and a two-fold higher likelihood of WNV in the age group 26 – 45 years relative to under 25 years (OR=2.27, 95% CI = 1.06–4.86, P value= 0.035). There was no observed effect of occupation on WNV exposure in this site.

## 4 Discussion

Globally, arboviruses such as yellow fever (YFV), dengue (DENV), West Nile (WNV) and Zika (ZIKV) have become a major public health threat with a significant socioeconomic burden (13, 15, 49). In sub-Saharan Africa, these infections mostly go undetected or misdiagnosed in high-risk remote endemic areas that are hard to reach due to inaccessible health facilities, infrastructure or personnel (7). This study analyzed the seroprevalence of yellow fever, dengue-2 virus, and related *Flaviviruses* (West Nile virus and Zika virus) circulating among the human population in Nguruman and Kerio Valley, Kenya using Plaque reduction neutralization test (PRNT). The plaque reduction neutralization test (PRNT) is considered the gold standard for identifying serological specificity among viruses and differentiation of Flavivirus infections (42, 43, 50, 51). We employed a more stringent PRNT_90_ which has a higher specificity and can reduce the background serum cross-reactivities across flaviviruses, hence it is very beneficial for epidemiological and diagnostic studies in dengue and yellow fever endemic areas (42, 51, 52). Thus, the method ruled out any cross-reactivities among the flaviviruses tested. Of the 480 samples that were analyzed, at least one of the four viruses was detected in 54.5% (262/480; 95% confidence interval [CI] 50.1–59.0%). The rates of exposure to these infections for every virus and site were found to be varied. The detected circulating viruses; YFV, DENV-2, WNV, and ZIKV were varied in proportion in both Nguruman and Kerio Valley. Therefore, the observed variation was associated with the varying exposure risk to these infections across sampled regions because of the wide vector distribution, diverse climatic and geographical conditions (2, 53).

Moreover, arboviral diseases are known to have wide non-specific clinical presentations and cross-reactivity (54, 55). These variations could also play a role in experiencing challenges in deciding an encountered differential diagnosis due to shared similar febrile clinical presentation illnesses by related viruses or cross-reactivity of these arboviruses in laboratory diagnosis (7, 56, 57). As a result, it is important to raise awareness among doctors and the general public about arbovirus infections as potential causes of febrile illnesses. Additionally, providing diagnostic tools at health facilities could aid in the identification and distinction of arboviral infections from other febrile conditions (54). Nevertheless, Kerio Valley is one of the regions in the country that has poor access to health facilities, infrastructure and personnel following cattle rustling and insecurity (58). Therefore, the unavailability of arboviral diagnostic capacity within the available health facilities could also limit tracking or conducting surveillance of these flaviviruses. Thus, the data from the study will be key in planning surveillance and diagnostic capabilities.

In this study, YFV-neutralizing antibodies with significantly higher rates were detected in Kerio Valley (145/286, 51%) compared to Nguruman (P<0.0001). The observed high rates of arbovirus exposure may be attributed to the prior mass yellow fever vaccination conducted following the YFV outbreak that occurred in Baringo in 1992/93 and subsequent routine YF immunization instituted for children at 9 months (17, 18). Regardless of the level of disease protection that could be maintained after three decades of routine immunization, Yellow fever infections could still occur if sufficient herd immunity is not attained. In addition, mosquito vectors associated with yellow fever transmission; *Aedes africanus* and *Aedes keniensis* are still circulating in Baringo County (18, 59, 60) and could still transmit this virus. This study further suggests the existence of a low-level active circulation of YFV in Nguruman with 6% neutralizing antibodies being detected. However, up to date, no documented outbreak of YFV in Nguruman has been reported. None of the participants in Nguruman were vaccinated against the yellow fever virus. Furthermore, economic activities like wild animals, forested conservancies and subsistence farming with intensive irrigation in Nguruman provide mosquito breeding grounds. The herders’ frequent travels in search of pasture and water in these forested conservancies increase and provide human-sylvatic mosquito contact hence this human activity of encroaching on forest ecosystems offers YFV a reservoir and a means of transmission (53). Additionally, human migration into urban areas, population growth, climate change, and changes in land use (such as deforestation) are all possible causes that could have increased *Aedes* mosquito numbers and aided transmission of the Yellow fever virus (2). Additionally, the majority of the flaviviruses are dependent on suitable environmental condition that favors the survival and distribution of their vectors and thus are likely to re-emerge in new locations with favorable conditions (61–63). Thus, our study shows an increased risk of continued sylvatic transmission coupled with the suitable ecological factors that could favor Flavivirus transmission, even in previously naïve regions like Nguruman. Thus, monitoring the risk of transmission of these flaviviruses should provide information for surveillance of flaviviruses in the future and preparedness for outbreaks in the area.

DENV-2 seropositivity was detected only in Nguruman. Dengue outbreak was first reported along the Kenyan coast in 1982 (12). Ever since, several positive cases have been detected suggesting a likelihood of the regional spread of the virus (49). In 2011, the dengue virus re-emerged, leading to outbreaks in the coastal and northern regions of Kenya (15, 64). In addition, low endemicity of Dengue infections has been reported confirming the occurrence of DENV in areas outside the initial geographical outbreak boundaries (15, 49, 56, 65). The climate change and a dryland ecosystem in Nguruman could favor a high abundance of *Aedes aegypti* mosquito vectors that could be contributing to the transmission of DENV. Other studies have shown that the primary dengue vector, *Aedes aegypti*, is widely distributed throughout the country confirming the underlying risk of Dengue outbreaks (38, 60, 66, 67). These findings may indicate the continued spread of dengue from the Kenyan coast where it has been more common, thus, there is a need for continuous Dengue serosurvey to avert possible outbreaks.

West Nile virus (WNV) neutralizing antibodies were detected in both study sites with high frequency (40.2%) being detected in Nguruman. The observed frequency was found to be higher than those previously detected in Turkana (10.2%) (2). The existence of a conservancy park, lakes and rivers in both Nguruman and Kerio Valley could be associated with high-frequency detected WNV following the provision of good breeding grounds for *Culex* mosquito species and the existence of diverse bird species, which are also important in WNV transmission cycles (2, 28, 68). The rapid adaptation of WNV to infect local mosquito vectors is responsible for the virus’s wider epidemiological dissemination (62).

Although there has never been a reported or confirmed outbreak of Zika in Kenya, our findings suggest the circulation of Zika in Nguruman and Kerio Valley counties. The prevalence of Zika was detected in the studied regions with almost the same proportions in both Kerio Valley (5%) and Nguruman (6%). Studies on human exposure to ZIKV in Kenya are low (2, 32, 33). The detected low proportion of Zika exposures could be attributed to inadequate clinical and laboratory facilities rendering the disease unnoticed/undetected. Both Nguruman and Kerio Valley have an abundance of mosquito vectors whereby human interaction with sylvatic mosquitoes with the transmission maintained from the frequent movement of residents in search of pasture, firewood or water. Furthermore, there have been proposals to attribute the rise of ZIKV infections in people to ecological reasons, which are a result of increased mosquito transmission by *Aedes species* (53). This ecosystem could be targeted for arboviral surveillance to prevent viral transmission that could result in outbreaks.

The seropositivity also varied by gender and age groups in both Kerio Valley and Nguruman. Females in Kerio Valley had significantly higher neutralizing antibodies than males. This reflects the enrollment rates in the study also concentrated in the same gender where more females 60.21% (n=289) than males 39.79% (n=191) were enrolled. The high enrollment of women may be influenced by the tendency of women tend to exhibit hospital-seeking behaviors as compared to men. Men being away from homes mostly grazing livestock and farming may influence their low level of enrollment. Men were also more likely to be infected with YFV and WNV than females. This could be explained by the activities males perform such as farming, looking for pasture and taking care of livestock whereby these kinds of outside activities predispose them to infectious mosquito bites. This could account for the glaring difference between the exposure rates of men and women within study areas.

Most of the participants (56.25%) were below the age of 25 years which mainly comprised school-going children. In Kerio Valley, seropositivity declined with age. Those below 25 years’ age group had higher neutralizing antibodies to YFV, with a similar pattern observed for WNV and ZIKV. In Nguruman, neutralizing antibodies against WNV were highest in the 26–45 years’ group with the odds of exposure being more in the same age group which may indicate low-level ongoing and persistent transmission of this virus in the study areas. Active virus transmission is supported by evidence of virus isolation from mosquitoes of Culex spp. in this ecosystem (38, 68).

This study had limitations. For instance, our study was focused only on a single serotype (DEN-2) indicating the need for an additional study on DENV genetic diversity to ascertain the precise prevalence of dengue in the studied region and its serotype distribution.

## 5 Conclusion

The findings from the study suggest that there is circulation of the *Flaviviruses;* YFV, WNV and Zika viruses of great public health importance in both Nguruman and Kerio Valley that could be contributing to under-recognized clinical disease. This study confirms the need to administer vaccines against these viruses to a level (80%) that could achieve herd immunity besides the implementation of vector control strategies to mitigate the potential risk of an outbreak.

## Supplementary Material

Table 1

Table 2

The Supplementary Material for this article can be found online at: https://www.frontiersin.org/articles/10.3389/fviro.2024.1459021/full#supplementary-material

## Figures and Tables

**TABLE 1:** Plaque assay results showing the calculated titers of DENV-2, YFV, ZIKV and WNV.

Virus	Passage Number	Number of plaques	Dilution factor	The volume of diluted virus added (μl)	Titer (pfu/ml)
DENV-2 (008/01/2012)	1	48	10^−2^	100	4.8X10^4^
YFV(YFXSMB)	2	82	10^−3^	100	8.2X10^5^
ZIKV(MR766)	3	38	10^−2^	100	3.8X10^4^
WNV(AMH005348)	2	53	10^−2^	100	5.3X10^4^

YFV, Yellow fever virus; WNV, West Nile virus, DENV-2, Dengue-2 virus; ZIKV, Zika Virus.

**TABLE 2:** Demographic characteristics of the study population from Nguruman in Kajiado and Kerio Valley in Baringo Counties.

CHARACTERISTIC	NGURUMAN		KERIO VALLEY		COMBINED	
	N	%	N	%	N	%
**ALL**	194	100	286	100	480	100
**SEX**						
**Male**	99	51.03	92	32.17	191	39.79
**Female**	95	48.97	194	67.83	289	60.21
**AGE GROUP (YEARS)**						
**25 and Below**	102	52.6	168	58.74	270	56.25
**26 – 45**	75	38.7	69	24.12	144	30
**46 – 66**	16	8.2	37	12.94	53	11.04
**66 and above**	1	0.5	12	4.2	13	2.71
**OCCUPATION**						
**Farmer**	100	51.55	78	27.27	178	37.16
**Student**	38	19.59	108	37.76	146	30.48
**Businessman**	4	2.06	11	3.85	15	3.13
**Business lady**	4	2.06	9	3.15	12	2.51
**Housewife**	16	8.25	61	21.33	77	16.08
**Didn’t indicate**	5	2.58	3	1.05	8	1.67
**Non**	27	13.92	16	5.59	43	8.98

**TABLE 3:** Prevalence of neutralizing antibodies against yellow fever, dengue-2, and related flaviviruses in Nguruman and Kerio Valley.

Virus	Nguruman n (%)	Kerio Valley n (%)	Combined n (%)
**YFV**	11 (6)	145 (51)^a^	156 (32.5)
**WNV**	52 (27)^b^	26 (9)	78 (16.25)
**ZIKV**	12 (6)	13 (5)	25 (5.2)
**DENV-2**	3 (2)	0 (0)	3 (1)

YFV, Yellow fever virus; WNV, West Nile virus, DENV-2, Dengue-2 virus; ZIKV, Zika Virus.

a Significant difference between the sites (p<0.0001) by the Chi-square test.

b Significant difference between the sites (p<0.0001) by the Chi-square test.

**TABLE 4:** A comparative analysis of the two most prevalent flaviviruses (YFV and WNV) in Nguruman (Kajiado County) and Kerio Valley (Baringo County) by age, gender and occupation using Multinomial Logistic regression model.

Variable	Kerio Valley				Nguruman			
	Yellow fever (YFV)		West Nile (WNV)		Yellow fever (YFV)		West Nile (WNV)	
	OR (95% CI)	p-value	OR (95% CI)	p-value	OR (95% CI)	p-value	OR (95% CI)	p-value
**SEX**								
Female	Reference		Reference		Reference		Reference	
Male	1.31(0.26–6.50)	0.7	2.19 (1.14–4.19)	0.018*	0.78 (0.01–46.2)	>0.9	2.49 (1.40–4.42)	0.002*
**AGE**								
25yrs & below	Reference		Reference		Reference		Reference	
26–45yrs	0.74 (0.13–4.16)	0.7	2.83 (1.13–7.12)	0.027*	0.81 (0.27–2.43)	0.7	2.27(1.06–4.86)	0.035*
46–65yrs	0.32 (0.03–3.64)	0.4	1.95 (0.66–5.72)	0.2	0.30 (0.03–2.73)	0.3	1.72 (0.66–4.45)	0.3
66yrs & above	0.00 (0.00–0.00)	<0.001	2.60 (0.45–15.0)	0.3	0.00 (0.00–0.00)	>0.9	2.28 (0.41–12.8)	0.3
**OCCUPATION**								
Business lady	Reference		Reference		Reference		Reference	
Businessman	0.32 (0.01–10.3)	0.5	0.22 (0.03–1.88)	0.2	0.42 (0.02–9.33)	0.6	0.24 (0.04–1.62)	0.14
Farmer	0.22 (0.01–3.93)	0.3	0.36 (0.06–2.09)	0.3	0.51 (0.05–5.25)	0.6	0.45 (0.10–1.94)	0.3
Housewife	0.98 (0.06–14.8)	>0.9	0.48 (0.08–2.93)	0.4	1.56 (0.15–16.3)	0.7	0.69 (0.15–3.15)	0.6
Didn’t indicate	0.00 (0.00–0.00)	<0.001	0.15 (0.01–3.15)	0.2	0.00 (0.00–0.00)	<0.001	0.19 (0.01–2.78)	0.2
Student	0.15 (0.01–2.11)	0.2	0.35 (0.06–1.95)	0.2	0.39 (0.03–4.09)	0.4	0.43 (0.10–1.85)	0.3

* Indicates statistically significant value.

## Data Availability

The original contributions presented in the study are included in the article/Supplementary Material. Further inquiries can be directed to the corresponding authors.

## Abbreviations:

CI: Confidence Interval
DENV: Dengue Virus
ELISA: Enzyme-Linked Immunoassay
OR: Odds ratio
PRNT: Plaque Reduction Neutralization Test
RT-PCR: Reverse Transcription Polymerase Chain Reaction
WHO: World Health Organisation
WNV: West Nile virus
YFV: Yellow fever virus
ZIKV: Zika Virus
