# Supplementary material for: Seroprevalence of dengue, yellow fever, and related flaviviruses among the rural human population in Nguruman and Kerio Valley, Kenya
Source: Front Virol. Author manuscript; Available in PMC 2026 Mar 19. (PMC12998444; doi:10.3389/fviro.2024.1459021)
Supplement: Table 1 [file NIHMS2113165-supplement-Table_1.docx]

**Table 1. Endpoint titres of the samples with neutralizing antibodies collected from Nguruman and analyzed using PRNT_90_**

| Neutralization antibody titre (90% Plaque reduction) | | | | | |
| --- | --- | --- | --- | --- | --- |
| Sample No. | **YFV** | **DENV** | **WNV** | **ZIKV** | **Inferred virus** |
| 034/ESP/01 | 1:20 |  |  |  | YFV |
| 034/ESP/04 | 1:80* |  | 1:20 |  | YFV |
| 034/ESP/07 |  |  | 1:40 |  | WNV |
| 034/ESP/10 |  |  | 1:80* | 1:20 | WNV |
| 034/ESP/11 |  |  |  | 1:20 | ZIKV |
| 034/ESP/13 | 1:20 |  | 1:160* |  | WNV |
| 034/ESP/20 | 1:20 |  |  |  | YFV |
| 034/ESP/22 |  |  | 1:160 |  | WNV |
| 034/ESP/25 |  |  | 1:160 |  | WNV |
| 034/ESP/29 | 1:20 |  | 1:160* |  | WNV |
| 034/ESP/36 | 1:20 |  | 1:80* |  | WNV |
| 034/ESP/40 |  |  | 1:160* | 1:20 | WNV |
| 034/ESP/41 |  |  | 1:20 |  | WNV |
| 034/ESP/44 |  |  | 1:80* | 1:20 | WNV |
| 034/ESP/45 |  |  | 1:40 |  | WNV |
| 034/ESP/46 |  |  | 1:80 |  | WNV |
| 034/ESP/47 | 1:20 |  | 1:160* |  | WNV |
| 034/ESP/48 | 1:80 |  |  |  | YFV |
| 034/ESP/53 |  |  | 1:40 |  | WNV |
| 034/ESP/59 | 1:20 |  | 1:320* | 1:40 | WNV |
| 034/ESP/63 | 1:20 |  | 1:160* |  | WNV |
| 034/ESP/67 | 1:20 |  | 1:80* |  | WNV |
| 034/ESP/73 | 1:20 |  |  |  | YFV |
| 034/ESP/74 | 1:80 |  | 1:320* | 1:20 | WNV |
| 034/ESP/75 |  |  | 1:80 |  | YFV |
| 034/ESP/76 | 1:80* |  | 1:20 |  | YFV |
| 034/ESP/79 |  |  | 1:20 |  | WNV |
| 034/ESP/80 |  |  | 1:20 |  | WNV |
| 034/ESP/81 |  |  | 1:20 |  | WNV |
| 034/ESP/94 |  |  | 1:160 |  | WNV |
| 034/ESP/95 |  |  | 1:80* | 1:20 | WNV |
| 034/ESP/98 |  |  | 1:20 |  | WNV |
| 034/ESP/100 |  |  | 1:320 |  | WNV |
| 034/ESP/102 |  |  |  | 1:20 | ZIKV |
| 034/ESP/104 |  |  | 1:160* | 1:20 | WNV |
| 034/ESP/105 | 1:80 |  |  |  | YFV |
| 034/ESP/106 |  |  | 1:20 |  | WNV |
| 034/ESP/108 |  |  |  | 1:20 | ZIKV |
| 034/ESP/109 |  |  |  | 1:20 | ZIKV |
| 034/ESP/112 | 1:20 |  |  | 1:80* | ZIKV |
| 034/ESP/114 | 1:20 |  |  |  | YFV |
| 034/ESP/120 |  |  | 1:20 | 1:160* | ZIKV |
| 034/ESP/122 |  |  | 1:80 |  | WNV |
| 034/ESP/123 |  |  | 1:20 |  | WNV |
| 034/ESP/125 |  |  | 1:320 |  | WNV |
| 034/ESP/130 | 20 |  |  |  | YFV |
| 034/ESP/132 | 1:20 |  | 1:20 | 1:80* | ZIKV |
| 034/ESP/135 |  | 1:20 |  |  | DENV |
| 034/ESP/137 |  |  | 1:80 |  | WNV |
| 034/ESP/139 |  |  | 1:320* | 1:40 | WNV |
| 034/ESP/140 |  |  | 1:20 | 1:160* | ZIKV |
| 034/ESP/141 |  |  | 1:320 |  | WNV |
| 034/ESP/143 |  |  | 1:160* | 1:20 | WNV |
| 034/ESP/144 |  |  | 1:80 |  | WNV |
| 034/ESP/145 |  |  |  | 1:40 | ZIKV |
| 034/ESP/146 |  |  | 1:2560 |  | WNV |
| 034/ESP/148 |  |  |  | 1:20 | ZIKV |
| 034/ESP/152 |  |  | 1:80 |  | WNV |
| 034/ESP/156 |  |  | 1:20 |  | WNV |
| 034/ESP/158 | 1:20 |  | 1:320* | 1:20 | WNV |
| 034/ESP/161 |  |  | 1:160 |  | WNV |
| 034/ESP/162 |  |  | 1:160 |  | WNV |
| 034/ESP/165 |  |  | 1:320 |  | WNV |
| 034/ESP/175 |  |  | 1:20 |  | WNV |
| 034/ESP/176 |  |  |  | 1:20 | ZIKV |
| 034/ESP/179 |  |  | 1:2560 |  | WNV |
| 034/KHS 01 |  |  | 1:320 |  | WNV |
| 034/KHS 05 |  |  | 1:40 |  | WNV |
| 034/KHS 06 |  |  | 1:160 |  | WNV |
| 034/KHS 07 |  |  | 1:40 |  | WNV |
| 034/KHS 08 | 1:80 |  |  |  | YFV |
| 034/KHS 09 |  |  | 1:160 |  | WNV |
| 034/KHS 14 |  |  | 1:320* | 1:40 | WNV |
| 034/KHS 15 |  | 1:40 |  |  | DENV |
| 034/KHS 16 |  |  |  | 1:40 | ZIKV |
| 034/KHS 21 |  | 1:80 |  |  | DENV |
| 034/KHS 22 |  |  | 1:1280 |  | WNV |
| 034/KHS 23 |  |  | 1:80 |  | WNV |

YFV, Yellow fever virus; DENV, Dengue Virus; WNV, West Nile virus; ZIKV, Zika Virus

*A PRNT_90_ titre of ≥20 and a 4-fold difference between YFV, WNV, and ZIKV titres
