# Supplementary material for: Seroprevalence of dengue, yellow fever, and related flaviviruses among the rural human population in Nguruman and Kerio Valley, Kenya
Source: Front Virol. Author manuscript; Available in PMC 2026 Mar 19. (PMC12998444; doi:10.3389/fviro.2024.1459021)
Supplement: Table 2 [file NIHMS2113165-supplement-Table_2.docx]

**Table 2. Endpoint titres of the samples with neutralizing antibodies collected from Kerio Valley and analyzed using PRNT_90_**

| Neutralization antibody titre (90% Plaque reduction) | | | | | |
| --- | --- | --- | --- | --- | --- |
| Sample No. | **YFV** | **DENV** | **WNV** | **ZIKV** | **Inferred virus** |
| 030/HM/001 | 1:80* |  | 1:20 |  | YFV |
| 030/HM/005 |  |  | 1:20 |  | WNV |
| 030/HM/006 | 1:20 |  | 1:80* |  | WNV |
| 030/HM/007 | 1:160 |  |  |  | YFV |
| 030/HM/008 | 1:160 |  |  |  | YFV |
| 030/HM/009 |  |  | 1280 |  | WNV |
| 030/HM/014 | 1:160 |  |  |  | YFV |
| 030/HM/015 | 1:160* |  | 1:40 |  | YFV |
| 030/HM/016 | 1:20 |  |  | 1:160* | ZIKV |
| 030/HM/017 | 1:20 |  |  |  | YFV |
| 030/HM/018 | 1:160* |  | 1:40 |  | YFV |
| 030/HM/021 | 1:160 |  |  |  | YFV |
| 030/HM/023 | 1:160 |  |  |  | YFV |
| 030/HM/024 | 1:160 |  |  |  | YFV |
| 030/HM/026 | 1:640 |  |  |  | YFV |
| 030/HM/027 | 1:20 |  |  |  | YFV |
| 030/HM/029 | 1:80 |  |  |  | YFV |
| 030/HM/030 | 1:80 |  |  |  | YFV |
| 030/HM/032 | 80* |  |  | 1:20 | YFV |
| 030/HM/033 | 640 |  |  |  | YFV |
| 030/HM/034 | 80 |  |  |  | YFV |
| 030/HM/035 | 80* |  |  | 1:20 | YFV |
| 030/HM/036 | 20 |  |  |  | YFV |
| 030/HM/038 |  |  |  | 1:20 | ZIKV |
| 030/HM/039 | 1:80 |  |  |  | YFV |
| 030/HM/040 | 1:80 |  |  |  | YFV |
| 030/HM/041 | 1:20 |  | 1:160* |  | WNV |
| 030/HM/042 |  |  | 1:40 |  | WNV |
| 030/HM/044 | 1:20 |  |  |  | YFV |
| 030/HM/046 | 1:20 |  |  |  | YFV |
| 030/HM/050 |  |  | 1:20 |  | WNV |
| 030/HM/053 |  |  |  | 1:20 | ZIKV |
| 030/HM/056 | 1:20 |  |  |  | YFV |
| 030/HM/058 | 1:20 |  |  |  | YFV |
| 030/HM/059 | 1:20 |  |  |  | YFV |
| 030/HM/063 | 1:40 |  |  |  | YFV |
| 030/HM/064 | 1:40 |  |  |  | YFV |
| 030/HM/066 | 1:80 |  |  |  | YFV |
| 030/HM/067 | 1:80 |  |  |  | YFV |
| 030/HM/069 | 1:20 |  |  |  | YFV |
| 030/HM/072 | 1:80 |  |  |  | YFV |
| 030/HM/075 | 1:20 |  |  |  | YFV |
| 030/HM/076 |  |  | 1:20 |  | WNV |
| 030/HM/078 | 1:80 |  |  |  | YFV |
| 030/HM/079 | 1:80 |  |  |  | YFV |
| 030/HM/081 | 1:80 |  |  |  | YFV |
| 030/HM/083 | 1:20 |  |  |  | YFV |
| 030/HM/085 | 1:20 |  |  |  | YFV |
| 030/HM/086 | 1:40 |  |  |  | YFV |
| 030/HM/087 | 1:20 |  |  |  | YFV |
| 030/HM/090 | 1:80 |  |  |  | YFV |
| 030/HM/093 | 1:20 |  | 1:40 | 1:160* | ZIKV |
| 030/HM/094 | 1:20 |  |  |  | YFV |
| 030/HM/095 | 1:20 |  |  |  | YFV |
| 030/HM/096 | 1:80 |  |  |  | YFV |
| 030/HM/097 | 1:20 |  |  |  | YFV |
| 030/HM/098 | 1:80 |  |  |  | YFV |
| 030/HM/099 | 1:80* | 1:20 |  |  | YFV |
| 030/HM/100 | 1:20 |  |  |  | YFV |
| 030/HM/101 | 1:20 |  |  |  | YFV |
| 030/HM/102 | 1:20 |  |  |  | YFV |
| 030/HM/103 | 1:20 |  |  |  | YFV |
| 030/HM/104 | 1:80 |  |  |  | YFV |
| 030/HM/105 | 1:80* |  | 1:20 |  | YFV |
| 030/HM/106 | 1:40 |  | 1:160* |  | WNV |
| 030/HM/108 | 1:20 |  |  |  | YFV |
| 030/HM/109 | 1:80 |  |  |  | YFV |
| 030/HM/110 |  |  | 1:20 |  | WNV |
| 030/HM/111 |  |  |  | 1:20 | ZIKV |
| 030/HM/113 | 1:80 |  |  |  | YFV |
| 030/HM/114 | 1:160 |  |  |  | YFV |
| 030/HM/115 | 1:160 |  |  |  | YFV |
| 030/HM/116 | 1:20 |  |  |  | YFV |
| 030/HM/117 | 1:320 |  |  |  | YFV |
| 030/HM/120 | 1:80* |  | 1:20 |  | YFV |
| 030/HM/121 | 1:640 |  |  |  | YFV |
| 030/HM/122 | 1:20 |  |  |  | YFV |
| 030/HM/123 | 1:40 |  |  |  | YFV |
| 030/HM/126 | 1:40 |  |  |  | YFV |
| 030/HM/127 | 1:160* |  | 1:20 |  | YFV |
| 030/HM/128 | 1:160* |  |  | 1:20 | YFV |
| 030/HM/130 | 1:40 |  |  |  | YFV |
| 030/HM/131 | 1:320* |  | 1:80 |  | YFV |
| 030/HM/133 | 1:40 |  |  |  | YFV |
| 030/HM/136 | 1:320 |  |  |  | YFV |
| 030/HM/137 | 1:320* |  | 1:40 |  | YFV |
| 030/HM/138 | 1:40 |  |  |  | YFV |
| 030/HM/140 | 1:80 |  |  |  | YFV |
| 030/HM/142 |  |  | 1:40 |  | WNV |
| 030/HM/143 | 1:80 |  |  |  | YFV |
| 030/HM/144 | 1:40 |  |  |  | YFV |
| 030/HM/146 |  |  |  | 1:20 | ZIKV |
| 030/HM/148 |  |  | 1:20 |  | WNV |
| 030/HM/151 |  |  | 1:20 |  | WNV |
| 030/HM/152 | 1:40 |  |  |  | YFV |
| 030/HM/153 |  |  |  | 1:20 | ZIKV |
| 030/HM/155 |  |  | 1:80* | 1:20 | WNV |
| 030/HM/156 | 1:80 |  |  |  | YFV |
| 030/HM/158 |  |  | 1:20 | 1:80* | ZIKV |
| 030/HM/159 |  |  | 1:40 |  | WNV |
| 030/HM/160 | 1:40 |  |  |  | YFV |
| 030/HM/162 | 1:40 |  |  |  | YFV |
| 030/HM/163 | 1:40 |  |  |  | YFV |
| 030/HM/164 | 1:80* |  |  | 1:20 | YFV |
| 030/HM/165 |  |  |  | 1:20 | ZIKV |
| 030/HM/166 | 1:40 |  |  |  | YFV |
| 030/HM/168 | 1:80 |  |  |  | YFV |
| 030/HM/169 |  |  |  | 1:40 | ZIKV |
| 030/HM/170 | 1:40 |  |  |  | YFV |
| 030/HM/171 | 1:40 |  |  |  | YFV |
| 030/HM/172 | 1:40 |  |  |  | YFV |
| 030/HM/174 |  |  |  | 1:20 | ZIKV |
| 030/HM/177 | 1:40 |  |  |  | YFV |
| 030/HM/178 | 1:80 |  |  |  | YFV |
| 030/HM/179 | 1:40 |  |  |  | YFV |
| 030/HM/182 | 1:80 |  |  |  | YFV |
| 030/HM/183 | 1:80 |  |  |  | YFV |
| 030/HM/184 | 1:20 |  | 1:160* |  | WNV |
| 030/HM/185 | 1:320 |  |  |  | YFV |
| 030/HM/186 | 1:20 |  |  |  | YFV |
| 030/HM/187 | 1:80 |  |  |  | YFV |
| 030/HM/190 | 1:40 |  |  |  | YFV |
| 030/HM/193 |  |  | 1:20 |  | WNV |
| 030/HM/194 | 1:40 |  |  |  | YFV |
| 030/HM/195 | 1:40 |  |  |  | YFV |
| 030/HM/196 |  |  | 1:20 |  | WNV |
| 030/HM/197 | 1:80* |  |  | 1:20 | YFV |
| 030/HM/198 | 1:160 |  |  |  | YFV |
| 030/HM/199 | 1:40 |  |  |  | YFV |
| 030/HM/200 | 1:40 |  |  |  | YFV |
| 030/HM/202 | 1:160* |  | 1:20 | 1:20 | YFV |
| 030/HM/203 | 1:40 |  |  |  | YFV |
| 030/HM/204 | 1:320* |  | 1:20 | 1:40 | YFV |
| 030/HM/205 | 1:40 |  |  |  | YFV |
| 030/HM/206 | 1:20 |  |  |  | YFV |
| 030/HM/208 | 1:1280 |  |  |  | YFV |
| 030/HM/212 | 1:320 |  |  |  | YFV |
| 030/HM/213 | 1:80 |  |  |  | YFV |
| 030/HM/218 | 1:320 |  |  |  | YFV |
| 030/HM/219 | 1:40 |  |  |  | YFV |
| 030/HM/220 | 1:40 |  |  |  | YFV |
| 030/HM/222 | 1:40 |  |  |  | YFV |
| 030/HM/226 | 1:40 |  |  |  | YFV |
| 030/HM/228 | 1:40 |  |  |  | YFV |
| 030/HM/232 | 1:40 |  |  |  | YFV |
| 030/HM/234 |  |  | 1:40 |  | WNV |
| 030/HM/235 | 1:40 |  |  |  | YFV |
| 030/HM/237 |  |  |  | 1:20 | ZIKV |
| 030/HM/238 | 1:40 |  |  |  | YFV |
| 030/HM/239 | 1:80* |  |  | 1:20 | YFV |
| 030/HM/240 | 1:320* |  |  | 1:20 | YFV |
| 030/HM/242 | 1:20 |  | 1:80* |  | WNV |
| 030/HM/243 | 1:20 |  |  |  | YFV |
| 030/HM/246 |  |  | 1:20 |  | WNV |
| 030/HM/248 | 1:40 |  |  |  | YFV |
| 030/HM/250 | 1:20 |  |  |  | YFV |
| 030/HM/254 | 1:640 |  |  |  | YFV |
| 030/HM/256 | 1:40 |  |  |  | YFV |
| 030/HM/257 |  |  | 1:20 |  | WNV |
| 030/HM/262 | 1:40 |  |  |  | YFV |
| 030/HM/264 | 1:40 |  |  |  | YFV |
| 030/HM/269 |  |  | 1:20 |  | WNV |
| 030/HM/271 | 1:40 |  |  |  | YFV |
| 030/HM/273 | 1:20 |  |  |  | YFV |
| 030/HM/274 | 1:80 |  |  |  | YFV |
| 030/HM/275 | 1;20 |  |  |  | YFV |
| 030/HM/276 | 1:160 |  |  |  | YFV |
| 030/HM/277 |  |  | 1:20 |  | WNV |
| 030/HM/279 | 1:40 |  |  |  | YFV |
| 030/HM/281 | 1:40 |  |  |  | YFV |
| 030/HM/292 | 1:80 |  |  |  | YFV |
| 030/HM/293 | 1:40 |  |  |  | YFV |
| 030/HM/296 | 1:40 |  |  |  | YFV |
| 030/HM/297 |  |  |  | 1:20 | ZIKV |
| 030/HM/299 | 1:40 |  |  |  | YFV |
| 030/HM/302 | 1:80* |  | 1:20 |  | YFV |
| 030/HM/304 |  |  | 1:320 |  | WNV |
| 030/HM/306 | 1:80* |  | 1:20 |  | YFV |
| 030/HM/307 | 1:40 |  |  |  | YFV |
| 030/HM/310 |  |  | 1:160* | 1:20 | WNV |
| 030/HM/314 | 1:40 |  |  |  | YFV |
| 030/HM/320 |  |  | 1:80 |  | WNV |
| 030/HM/321 | 1:40 |  |  |  | YFV |
| 030/HM/322 | 1:40 |  |  |  | YFV |

YFV, Yellow fever virus; DENV, Dengue Virus; WNV, West Nile virus; ZIKV, Zika Virus

*A PRNT_90_ titre of ≥20 and a 4-fold difference between YFV, WNV, and ZIKV titres
